# Pseudogenes as Potential Diagnostic, Prognostic and Therapeutic Biomarkers in Colorectal Cancer: A Systematic Review

**DOI:** 10.1002/cnr2.70263

**Published:** 2025-06-24

**Authors:** Ali Asadzadeh, Alireza Zangooie, Parmida Bagheri, Helia Zangooie, Zahra Salehi

**Affiliations:** ^1^ Department of Biology Payame Noor University Shahre Rey Iran; ^2^ Cellular and Molecular Research Center Birjand University of Medical Sciences Birjnad Iran; ^3^ Student Research Committee Birjand University of Medical Sciences Birjand Iran; ^4^ Department of Biotechnology Faculty of Life Sciences and Biotechnology, Shahid Beheshti University Tehran Iran; ^5^ Student Research Committee Mashhad University of Medical Sciences Mashhad Iran; ^6^ Hematology, Oncology and Stem Cell Transplantation Research Center Research Institute for Oncology, Hematology and Cell Therapy, Tehran University of Medical Sciences Tehran Iran

**Keywords:** colorectal cancer, gene regulation, pseudogene, tumorigenesis

## Abstract

**Background:**

Colorectal cancer (CRC) is a leading cause of oncologic death worldwide. Elucidating the molecular mechanisms that underpin its pathogenesis is essential for identifying novel therapeutic targets. Pseudogenes have historically been regarded as non‐functional genomic vestiges but have gained recognition for their contributory roles in the oncogenesis of CRC.

**Aims:**

This systematic review aims to comprehensively evaluate the current literature regarding the involvement of pseudogenes in CRC, with a focus on their clinical relevance as diagnostic and prognostic biomarkers and their potential as innovative therapeutic targets.

**Methods and Results:**

We conducted a comprehensive literature search following the PRISMA guidelines across PubMed, SCOPUS, and Web of Science databases. Two reviewers independently carried out the screening of studies and extraction of relevant data. Nineteen studies met the inclusion criteria. These studies highlight pseudogenes' emerging role in colorectal cancer, transitioning from being seen as evolutionary remnants to recognized contributors in tumorigenesis. Key pseudogenes like DUXAP8, SUCLG2P2, and SUMO1P3 are linked to crucial CRC processes such as proliferation, migration, invasion, and angiogenesis. The diagnostic and prognostic potential is found in pseudogenes like MYLKP1 (with SNPs rs12490683 and rs12497343) and POU5F1P1 (SNP rs6983267). Additionally, CDCP1, SUCLG2P2, and MT1DP offer prognostic insights, guiding personalized treatment approaches. Pseudogenes such as CTNNAP1, NMRAL2P, and DUXAP8 show therapeutic potential, advocating for further research into their mechanisms to enhance CRC diagnostics and personalized care. The intricate involvement of pseudogenes in CRC pathogenesis underscores their importance.

**Conclusion:**

Our review illuminates the promise of pseudogenes as biomarkers and therapeutic targets, indicating a significant step toward the integration of pseudogenes in the future paradigm of precision medicine for CRC.

**Abbreviations**
CDCP1: CUB domain containing protein 1
ceRNA: competing endogenous RNA
CRC: colorectal cancer
CTNNAP1: contactin associated protein 1
CYP2A7: cytochrome P450 family 2 subfamily A member 7
DE: differentially expressed
DUXAP10: double homeobox A pseudogene 10
DUXAP8: double homeobox A pseudogene 8
EMT: epithelial‐mesenchymal transition
esiRNAs: endogenous small interfering RNAs
EZH2: enhancer of zeste 2 polycomb repressive complex 2
HCC: hepatocellular carcinoma
miRNA: microRNA
MLL3: mixed‐lineage leukemia protein 3
NMRAL2P: NmrA like redox sensor 2, pseudogene
NSCLC: non‐small cell lung cancer
PCNAP1: Proliferating Cell Nuclear Antigen Pseudogene 1
PKM2: Pyruvate kinase M2
Pseudogene: AOC4P amine oxidase copper containing 4
ROCK1: rho associated coiled‐coil containing protein kinase 1
SNPs: single nucleotide polymorphisms
SUCLG2: succinate‐CoA ligase GDP‐forming subunit beta
SUCLG2P2: succinate‐CoA ligase GDP‐forming beta subunit pseudogene 2
SUMO1P3: small ubiquitin‐like modifier 1 pseudogene 3
TCGA: The Cancer Genome Atlas Program

## 1 Introduction

In 2020, colorectal cancer (CRC) ranked as the third most frequently diagnosed cancer globally, with over 1.9 million new cases. It was the second leading cause of cancer‐related deaths, accounting for approximately 900 000 fatalities. It is predicted that CRC incidence could increase by 60% by 2030, with an estimated 3.2 million affected patients by 2040 [1, 2, 3]. Colorectal carcinogenesis is a complex biological process arising from the dysregulation of numerous cancer‐related genes. Consequently, a comprehensive understanding of the molecular mechanisms governing the development and progression of CRC is imperative for devising targeted strategies to alleviate the disease burden. Molecular biomarkers, pivotal for diagnosis and prediction, hold substantial clinical significance in the context of advancing precision medicine [4, 5].

Within the vast expanse of human DNA, the majority (98%) comprises non‐coding DNA, indicating genetic material that does not undergo translation into proteins. Among the diverse categories of non‐coding DNA, pseudogenes emerge as replicas of protein‐coding genes that have experienced multiple modifications, rendering them incapable of producing the originally encoded proteins. Despite their incapacity to generate functional proteins, contemporary research has brought to light the participation of pseudogenes in cancer [6]. Three distinct classifications of pseudogenes exist: processed, unprocessed, and unitary pseudogenes. Processed pseudogenes arise from the retrotransposition of mature mRNA molecules back into the genome. This retrotransposition involves the conversion of mRNA into DNA through reverse transcription, followed by integration into a novel genomic position, frequently on a distinct chromosome. Unprocessed pseudogenes, on the other hand, originate from the duplication of entire genomic regions, encompassing both exons and introns, of a functional gene. This duplication event may occur through mechanisms such as tandem duplications or unequal crossing over. Unitary pseudogenes arise from functional genes due to mutational events leading to the loss of their functionality. These mutations may involve frame‐shift mutations, premature stop codons, or extensive deletions, ultimately eliminating their capacity for protein coding [6, 7, 8].

Pseudogenes exhibit diverse functional roles, as exemplified by Ni et al., who illustrated that pseudogene TDGF1P3 acts as a competing endogenous RNA (ceRNA) by binding to miR‐338‐3p. This binding prevents miR‐338‐3p from interacting with its target mRNA pyruvate kinase M2 (PKM2), leading to increased CRC cell proliferation and invasion through upregulation of PKM2 [9]. Mechanistically, the pseudogene DUXAP8 in non‐small cell lung cancer (NSCLC) represses tumor suppressors EGR1 and RHOB by recruiting histone demethylase LSD1 and histone methyltransferase EZH2, promoting cell proliferation, migration, and invasion [10]. The lncRNA derivative of DUXAP8 in CRC interacts with EZH2 and H3K27me3 in the E‐cadherin promoter region, suppressing E‐cadherin expression and affecting cell proliferation, epithelial‐mesenchymal transition (EMT), and apoptosis [11]. These two are examples of how pseudogene and its derivative lncRNA could regulate gene expression epigenetically. In hepatocellular carcinoma (HCC), actively transcribed pseudogenes, particularly 𝜓PPM1K, produce endogenous small interfering RNAs (esiRNAs) that regulate protein‐coding genes. This study highlighted the regulatory impact of siRNAs derived from 𝜓PPM1K, downregulating NEK8 expression and inhibiting cell growth [12]. Additionally, in HCC, a gene conversion event between CYP2A6 and its pseudogene (CYP2A7) results in the CYP2A61B polymorphism, associated with increased stability and enzyme activity. Homozygosity for CYP2A61B is linked to higher cigarette consumption, potentially elevating lung cancer risk due to enhanced nicotine metabolism. Pseudogenes have thus been implicated in modifying chromatin structure through their transcripts or DNA sequences, contributing to cancer‐related processes [6, 13, 14]. This evidence demonstrated that pseudogenes could be diagnostic, prognostic, and therapeutic targets for cancers such as CRC. The objective of this systematic review is to investigate the role of pseudogenes in CRC.

## 2 Materials and Methods

### 2.1 Protocol and Registration

The present systematic review was conducted in accordance with the PRISMA guidelines, and the PICO (Population, Intervention, Comparator, and Outcomes) format was used to construct the scientific question of the present study. The review was conducted according to the protocol registered on the International Prospective Register for Systematic Reviews (PROSPERO Code: CRD42024512591).

### 2.2 Objectives and Scientific Question

The primary aim of this study was to evaluate the role and functions of pseudogenes in patients with CRC. Specifically, we sought to determine the clinical significance of these pseudogenes as diagnostic and prognostic biomarkers, as well as potential therapeutic targets.

### 2.3 Literature Search Methodology

For the literature search, three databases—PubMed, SCOPUS, and Web of Science—were employed to identify pertinent studies on 29 Oct 2023. The search strategy involved utilizing relevant MeSH terms for PubMed and a range of specific terms for SCOPUS and Web of Science, including “Colorectal Neoplasm,” “Colorectal Tumor,” “Colorectal Cancer,” “Colorectal Carcinoma,” “Pseudogene,” “Processed Gene,” “beta‐Tubulin Pseudogene,” and “beta Tubulin Pseudogene.” Detailed search queries and the number of studies identified from each database are presented in Table S1. To enhance the reliability of the search process and reduce the possibility of bibliographic exclusion, a cross‐database overlap check was implemented. This involved comparing the results retrieved from the three databases to identify duplicate records and ensure inclusion of unique and relevant studies across all platforms. Additionally, reference lists of included articles were manually screened to capture any potentially missed studies. This multi‐layered approach strengthens the reproducibility and comprehensiveness of the literature review.

### 2.4 Eligibility Criteria for Inclusion and Exclusion

Eligible studies included those that focused on patients with CRC or used CRC patient‐derived cell lines, with the primary objective of evaluating pseudogenes. Research that aimed at developing protocols for either screening pseudogenes or removing their detection in molecular diagnostic tests, as well as in vivo and in vitro experiments employing animal models and/or commercial human cell lines, non‐original articles (comprising all types of reviews, case reports/series, letters, editorials, etc.), papers in languages other than English, and papers for which full‐text access remained unavailable, even after an attempt to reach out to the primary author, were disqualified. Additionally, no year‐based filters were used, enabling the inclusion of all relevant studies from their start date to the 29 Oct 2023.

### 2.5 Literature Screening and Data Extraction

The literature screening process underwent a detailed two‐phase evaluation by two reviewers (PB and AA) to evaluate and choose papers based on previously defined eligibility criteria. The initial phase involved reviewing titles and abstracts, followed by a second phase of full‐text examination. Studies not meeting the criteria were excluded using predefined labels aligned with the exclusion criteria. The chosen studies proceeded to the data extraction phase, during which three reviewers (PB, AA, and AZ) gathered bibliographic information such as study title, primary author's name, publication year, and study design. Any disagreements were resolved through discussion, and a fourth reviewer (ZS) was consulted if discrepancies remained unresolved.

## 3 Results

### 3.1 Characteristics of Included Studies

**FIGURE 1:**
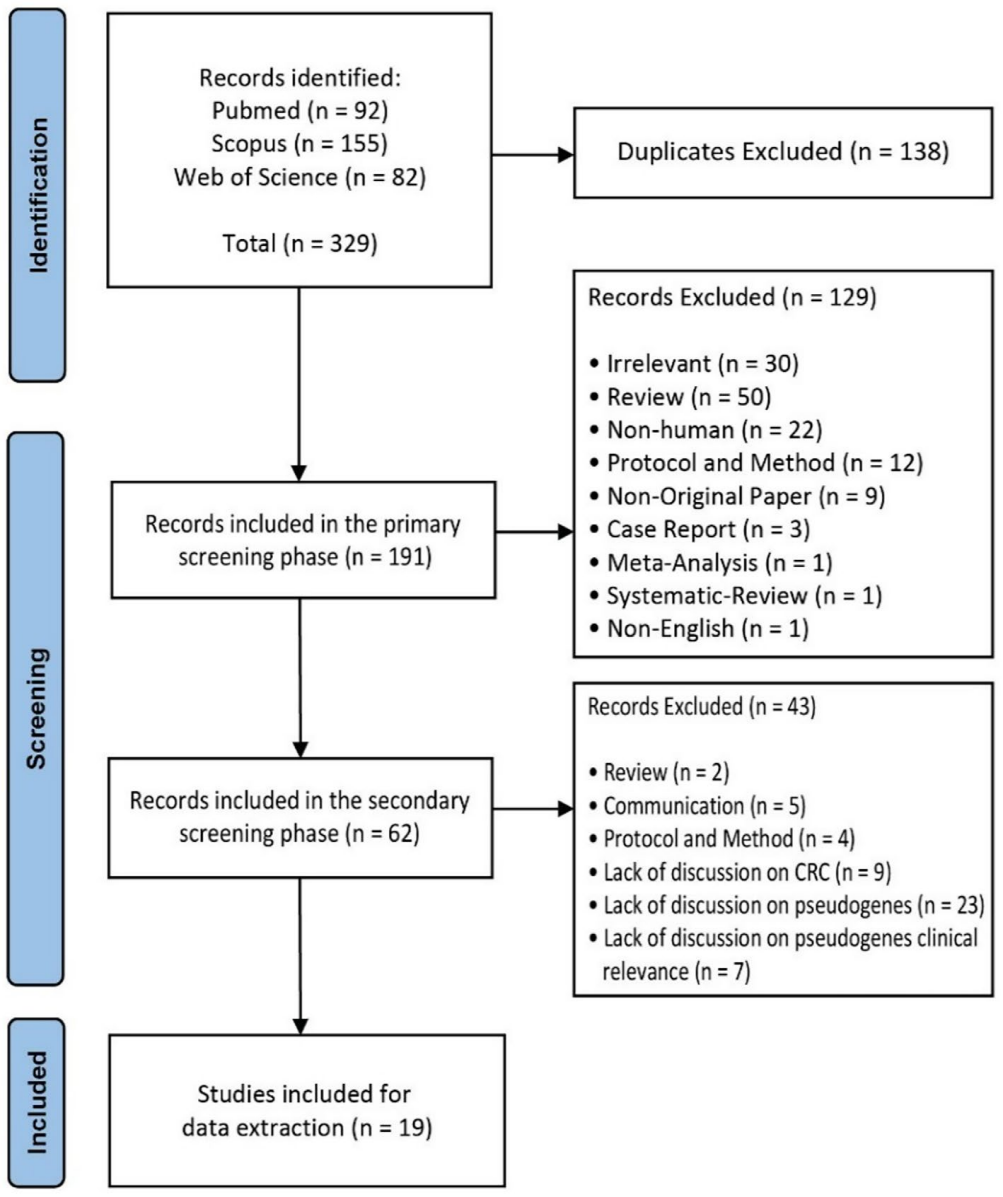
PRISMA flow chart of the present systematic review.

A total of 329 records were initially identified, of which 138 were duplicates and removed. After a detailed review process, 172 studies were further excluded, resulting in 19 studies being selected for final data extraction (Table S2). The selection workflow is depicted in the PRISMA flow chart shown in Figure 1.

In our article, 19 studies encompassing 3270 CRC patients from various countries were analyzed, with the majority conducted in China (63%), followed by the USA (12%), and with Iran, Italy, Sweden, and Taiwan each contributing 5% of the studies. Among these, 11 studies highlighted pseudogenes with prognostic significance, 3 identified diagnostic pseudogenes, and 6 proposed pseudogenes as potential therapeutic targets in CRC (Table 1).

**TABLE 1:** Functional and clinical information on the pseudogenes in the present study.

Pseudogene	Related cancer	Location of chromosome	Disorder	Type of pseudogene	Description/function	References
CTNNA1P1	Colorectal cancer	5q22.3	Isolation and characterization of a human pseudogene (CTNNAP1) for alpha E‐catenin (CTNNA1): assignment of the pseudogene to 5q22 and the alpha E‐catenin gene to 5q31. Downregulated pseudogene CTNNAP1 promote tumor growth in human cancer by downregulating its cognate gene CTNNA1 expression. Structure, expression and chromosome assignment of the human catenin (cadherin‐associated protein) alpha 1 gene (CTNNA1).	Therapeutic	Response to tenofovir Body weight gain Body mass index Self‐injurious behavior Late‐onset alzheimers disease	[15]
NMRAL2P	Liver, colon and prostate cancer	3q27.2	Gallbladder Cancer Lung Cancer	Therapeutic	Body height Age at menarche Body mass index Neurofibrillary tangles measurement Educational attainment	[16]
DUXAP8	Colorectal cancer	22q11.1	Renal Cell Carcinoma, Nonpapillary Gastric Cancer	Therapeutic		[11, 17, 18]
DUXAP10	Non‐small cell lung cancer (NSCLC), colorectal cancer	14q11.2	Ovarian Cancer Colorectal Cancer	Diagnostic, prognostic, or therapeutic	Attempted suicide	[5]
PCNAP1	Colorectal cancer	4q23	NA	Diagnostic	Alcohol consumption measurement Alcohol use disorder measurement Social interaction measurement Protein measurement Alcohol dependence measurement	[19]
ROCK1	Colorectal cancer	18q11.1	Breast Cancer Lung Cancer	Prognostic	Protein kinase which is a key regulator of the actin cytoskeleton and cell polarity Promotes keratinocyte terminal differentiation Plays a role in terminal erythroid differentiation Acts as a negative regulator of VEGF‐induced angiogenic endothelial cell activation	[20]
RPLP0P2	Colorectal cancer	11q12.2	Lung cancer susceptibility	Prognostic	Gamma‐linolenic acid measurement cc16 measurement Omega‐3 polyunsaturated fatty acid measurement Omega‐6:omega‐3 polyunsaturated fatty acid ratio Chronic obstructive pulmonary disease	[21]
PsiTPTE22	Colorectal cancer	22q11.1	NA	Diagnostic	Celiac disease	[22]
MYLKP1	Colorectal cancer	3p12.3	NA	Diagnostic	Visual perception measurement qt interval	[23]
SUCLG2P2	Colorectal cancer	12q22	NA	Prognostic	Acute myeloid leukemia Adolescent idiopathic scoliosis Neuritic plaque measurement Reading and spelling ability Reading	[24]
AOC4P	Colorectal cancer	17q21.31	Hepatocellular Carcinoma Colorectal Cancer	Prognostic	Body height Level of membrane primary amine oxidase in blood serum Type 2 diabetes mellitus c‐reactive protein measurement	[25]
PER3P1	Colorectal cancer	7p21.3	NA	Prognostic Diagnostic	phf‐tau measurement Neurofibrillary tangles measurement Body height Postural instability Protein measurement	[26]
MT1DP	Colorectal cancer	16q13	Hepatocellular carcinoma gastric cancer	Prognostic	High density lipoprotein cholesterol measurement Free cholesterol to total lipids in large vldl percentage Triglycerides to total lipids in large vldl percentage Sexual dimorphism Measurement	[27]
SUMO1P3	Colorectal cancer	1q23.2	Bladder Cancer Gastric Cancer	Prognostic	Dynamic nuclei (hole, folded or small irregular) Increased transferrin (TF) endocytosis Effect on mitosis	[28]
NCF1C	Colorectal cancer	7q11.23	NA	Prognostic	Enables superoxide‐generating NADPH oxidase activator activity Enables phosphatidylinositol binding	[4]
RP9P	Colorectal cancer	7p14.3	Retinitis Pigmentosa 9 Retinitis Pigmentosa	Prognostic	Red blood cell distribution width Orotate measurement Body height Serum metabolite measurement Uridine diphosphate measurement	[4]
DDX12P	Colorectal cancer	12p13.31	Warsaw Breakage Syndrome	Prognostic	Blood protein measurement Protein measurement Lymphocyte count Lymphocyte:monocyte ratio Lymphocyte percentage of leukocytes	[4]
PLEKHA8P1	Colorectal cancer	12q12	NA	Prognostic	Central precocious puberty Response to tenofovir Response to tenofovir	[4]
PPBPP2	Colorectal cancer	4q13.3	NA	Prognostic	Neutrophil count Myeloid white cell count Leukocyte count Basophil count Eosinophil count	[29]
RAB9BP1	Colorectal cancer	5q21.2‐q21.3	NA	Prognostic	Smoking initiation Body height Potassium measurement Educational attainment Sodium measurement	[29]
ANKRD20A9P	Colorectal cancer	13q11	NA	Prognostic	Longevity Remission Hepatitis c virus infection Response to allopurinol Uric acid measurement	[29]
CXADRP2	Colorectal cancer	15q11.2	NA	Prognostic		[29]
CTAGE11P	Colorectal cancer	13q22.2		Prognostic	Body height Heart failure Gut microbiome measurement rs‐10‐hydroxywarfarin measurement Susceptibility to pneumonia measurement	[29]
IFNA22P	Colorectal cancer	9p21.3	NA	Prognostic	Body height Acute myeloid leukemia Systemic lupus erythematosus Response to mepolizumab	[29]
LOC407835	Colorectal cancer	7q32.1	NA	Prognostic	Hippocampus volume change measurement Age at assessment	[29]
UQCRBP1	Colorectal cancer	Xp11.21	NA	Prognostic		[29]
ST13P4	Colorectal cancer	13q14.2	NA	Prognostic	bmi‐adjusted hip circumference Diastolic blood pressure Multiple sclerosis	[29]
HMGB3P1	Colorectal cancer	20q11.22	NA	Prognostic	Sexual dimorphism Measurement Heel bone mineral density bmi‐adjusted hip circumference Reticulocyte count	[29]
TOP1P1	Colorectal cancer	1q24.3	NA	Prognostic	Cysteinylglycine measurement x‐21842 measurement Type 2 diabetes mellitus	[29]

*Note:* The data provided by Systematic review and also GeneCards (https://www.genecards.org/).

### 3.2 Pseudogenes Functions and Mechanisms in CRC

Previously, pseudogenes were dismissed as evolutionary byproducts without function. Yet, the advancement of molecular biology has unveiled their significant roles in regulating biological activities, reshaping our understanding of their importance in the genome [11]. Studies have highlighted the significance of pseudogenes in affecting the invasive and migratory capabilities of CRC cells.

In a recent study, it was discovered that ROCK1 serves as a downstream target regulated by CDCP1, which in turn enhances the metastatic capabilities of CRC cells. ROCK1, located in 18q11.1, is a member of the serine/threonine kinases family, and CDCP1 was previously suggested to possibly play a role in promoting cancer metastasis. Hence, it was suggested that CDCP1 modulation of ROCK1 expression and the subsequent alterations in ROCK1 targets could be mechanisms driving CRC cell migration and invasion under the influence of CDCP1. Furthermore, CRC patients with high TNM stages exhibited high ROCK1 mRNA expression [20].

Furthermore, the evaluation of CRC patients defined the upregulation of DUXAP8 in tumor samples compared to normal tissues. The upregulation of DUXAP8, located in 22q11.1, enhanced cell growth in CRC cells in vitro, whereas silencing DUXAP8 triggered apoptosis and decreased invasion ability. DUXAP8‐silenced cells showed higher levels of E‐cadherin expression and lower vimentin, snail, and N‐cadherin expression levels, suggesting that DUXAP8 could induce the EMT process in CRC cells and migration. Further assays showed that DUXAP8 knockdown resulted in the inhibition of EZH2 and H3K27me3 binding to the E‐cadherin promoter, suggesting that DUXAP8 interacts with EZH2, contributing to E‐cadherin levels decrease and CRC cell proliferation [11]. DUXAP8 directly interacts with miR‐577 through its binding to the 3′‐UTR, resulting in a reciprocal relationship between miR‐577 and DUXAP8 levels. This interplay influences the expression of RAB14, a significant oncogene, thus altering the metastatic potential of CRC cells. Experiments involving DUXAP8 knockdown demonstrated its ability to impede cell proliferation, migration, and invasion in CRC cells, suggesting its potential role in modulating metastatic potential and impacting CRC development through its effects on cellular proliferation and apoptosis [17]. DUXAP8 promotes CRC progression by upregulating ZEB1 through competitive binding with miR‐519b‐3p. As a transcription factor, ZEB1 plays a crucial role in driving epithelial‐mesenchymal transition (EMT), facilitating tumor invasion and metastasis. Its elevated expression enhances CRC cell proliferation, migration, and invasive potential [30, 31, 32].

Another report found that the largest isoform of MLL3, a tumor‐suppressor gene, is transcribed from a promoter associated with a CpG island, which shares significant homology with a pseudo‐gene located on chromosome 22, termed psiTPTE22. Moreover, the CpG island in the promoter region of the pseudogene psiTPTE22 is densely methylated in primary CRCs and correlates with aging in normal epithelium [22]. SUCLG2P2, located on chromosome 12 with a transcript length of 1296 bp and one exon, exhibits significant homology to SUCLG2, a gene involved in the tricarboxylic acid cycle. SUCLG2P2 downregulation alongside SUCLG2 in colon cancer implies a shared mechanism in carcinogenesis. Although the precise mechanism remains undisclosed, SUCLG2P2 might operate as a competitive endogenous RNA (ceRNA), modulating gene expression by sequestering miRNAs and competing for miRNA binding. Moreover, given its resemblance to SUCLG2, SUCLG2P2 could regulate SUCLG2 expression, affecting cellular metabolism. Reducing SUMO1P3 levels in animal models leads to decreased tumor growth, metastasis, and changes in key protein expression patterns. The mechanism of SUMO1P3 in colon cancer involves regulating cell proliferation, modulating cell cycle progression, influencing key oncogenic pathways, and promoting angiogenesis [28].

**FIGURE 2:**
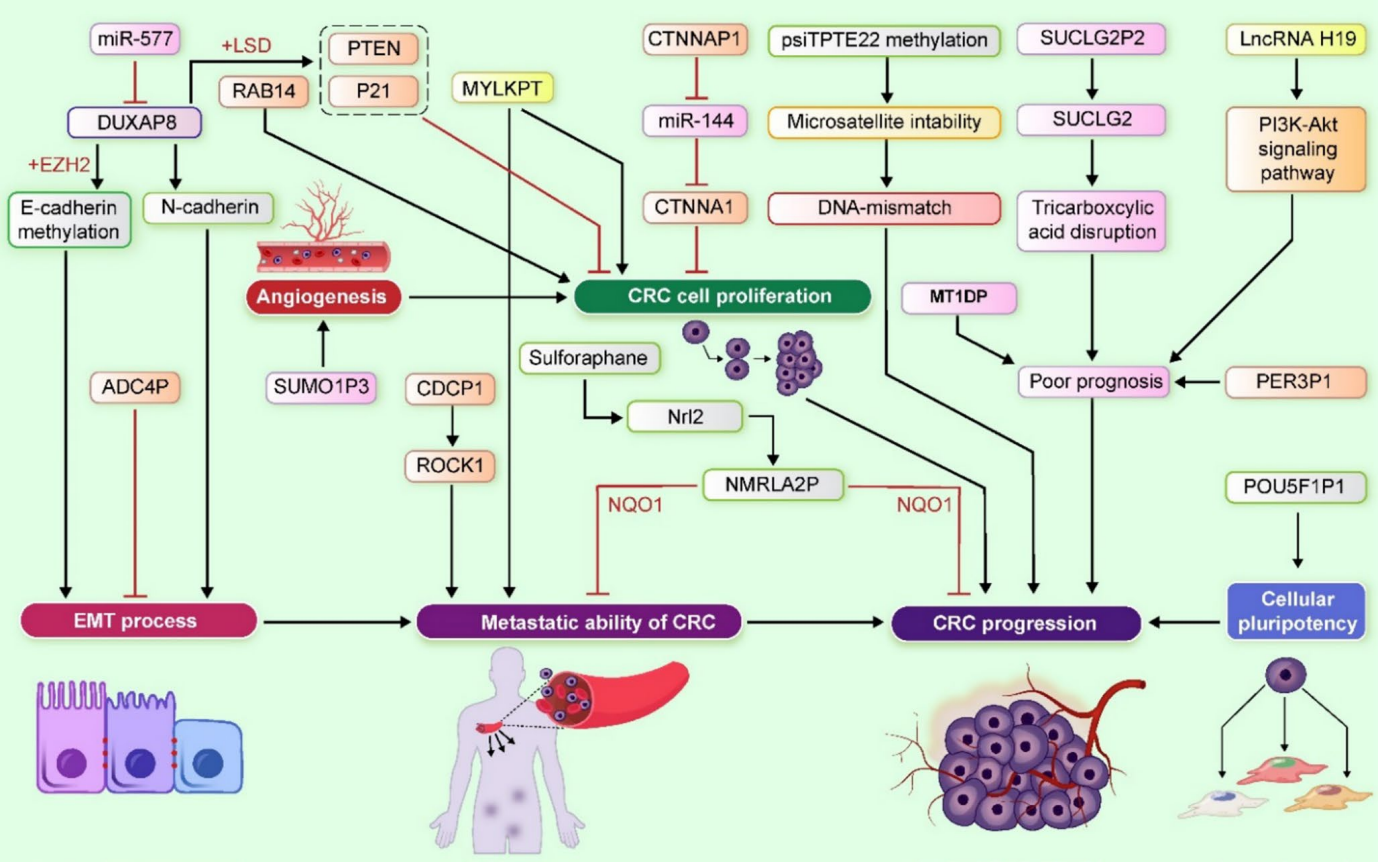
The functions and mechanisms of pseudogenes in CRC. The reviewed pseudogenes, including DUXAP8, SUCLG2P2, and SUMO1P3, play key roles in crucial activities in CRC, such as cell proliferation, migration, invasion, and angiogenesis. Pseudogenes like MYLKP1 and POU5F1P1 are involved in CRC metastasis and cellular pluripotency, having both diagnostic and prognostic implications. Additionally, CDCP1, SUCLG2P2, and MT1DP are significant prognostic biomarkers. Furhermore, CTNNAP1, NQO1, and DUXAP8 contributes to CRC progression by inhibiting miR‐144, interacting with NQO1, and affecting the EMT process along with interaction with LSD1, respectively. Therefore, These pseudoegenes can present potential therapeutic targets.

Also, gene expression pattern analysis of CTNNAP1 pseudogene in CRC and matching normal tissue pairs, revealed CTNNAP1 downregulation in 70% of tumors. A significant correlation was observed between CTNNAP1 expression levels and TNM staging, with advanced stages showing lower expression. CTNNAP1 expression level was also positively correlated with its cognate gene, CTNNA1. This pseudogene functions as a ceRNA, orchestrating the maintenance of CTNNA1 expression by engaging in a sophisticated competition for specific miRNA binding sites. The research highlighted that CTNNAP1 impacts CTNNA1 expression by acting as a decoy for miRNA‐141. In vitro findings revealed that miRNA‐141 overexpression reduced CTNNA1 mRNA, an effect reversed by CTNNAP1 introduction and miRNA‐141 knockdown. Additionally, miRNA‐141 was shown to shorten the half‐life of CTNNAP1 and CTNNA1, suggesting a suppressive role in these genes. CTNNAP1 upregulation inhibited cell growth, colony formation, and induced cell cycle arrest. In vitro overexpression of CTNNAP1 and CTNNA1 also inhibited CRC tumorigenesis, indicating their therapeutic potential in CRC [15]. In another report, Knockdown of DUXAP10 manifested a notable inhibition of cell proliferation, induction of cell apoptosis, and a significant increase in the G0/G1 cell population. Furthermore, the silencing of DUXAP10 demonstrated inhibitory effects on tumor growth in in vivo experiments. A mechanistic exploration indicated that DUXAP10, by interacting with LSD, facilitated CRC cell growth and suppressed cell apoptosis by downregulating the expression of p21 and PTEN, both recognized tumor suppressors. The findings suggest that the pseudogene derived from the lncRNA DUXAP10 plays a promotive role in the biological progression of CRC. Consequently, it emerges as a potential therapeutic target for interventions in CRC [5] (Figure 2).

### 3.3 Pseudogenes as Colorectal Cancer Markers

Given the investigation into pseudogenes roles in CRC tumorigenesis and their operational mechanisms within CRC cells (See Table 1 for more details), these elements have the potential to serve as biomarkers for CRC. This understanding paves the way for utilizing pseudogenes in diagnostic and prognostic applications as well as therapeutic targets in patients with CRC.

### 3.4 Prognostic

Pseudogenes have the potential to serve as biomarkers for predicting the clinical outcomes of CRC patients. A study highlighted the identification of 17 upregulated pseudogenes among the differentially expressed genes associated with poor prognosis in CRC patients [29]. Through an analysis of expression profiles in colon adenocarcinoma sourced from The Cancer Genome Atlas (TCGA), 31 differentially expressed (DE) pseudogenes, along with 17 DE miRNAs and 152 DE mRNAs were identified. Subsequently, the ceRNA network was constructed based on these RNA entities. Kaplan–Meier analysis revealed that 7 pseudogenes, 4 miRNAs, and 30 mRNAs displayed significant associations with overall survival. The ceRNA‐related DE pseudogenes underwent multivariate Cox regression analysis, leading to the identification of a 5‐pseudogene signature with the highest prognostic value for CRC. Validation of the results was carried out using the Gene Expression Omnibus database, along with quantitative real‐time PCR conducted on 113 pairs of CRC tissues and colon cancer cell lines [4]. The pseudogene PER3P1, a variant of the PER3 gene, along with the other period (PER) family (PER1/2/3), was studied for its prognostic value. It was found that PER3P1 expression did not have a significant impact on the overall survival (OS). Gene expression analysis showed that PER3P1, among all PER family genes, exhibited the greatest reduction in expression in tumor tissues, with a decrease of 77% compared to control samples. Additionally, a positive correlation was observed between CRC patients' age and PER3P1 pseudogene expression levels [26]. The lncRNA AOC4P was observed to be downregulated in CRC tissues and cell lines. The reduced expression of AOC4P is closely associated with advanced tumor stage and distant metastasis. Kaplan–Meier analysis demonstrated that CRC patients with low AOC4P expression experienced poorer overall survival compared to those with high AOC4P expression. In vitro assays further revealed that AOC4P overexpression significantly inhibited the proliferation, migration, and invasion of CRC cells. Additionally, AOC4P overexpression was found to suppress the EMT phenotype in CRC cells [25]. In another report, high expression of DUXAP8 was found to predict poor prognosis in colon cancer [18]. Moreover, this pseudogene was found to be overexpressed in higher stages of colon cancer (stage III and IV) and its increased expression in CRC correlates with advanced clinical progression and diminished survival rates among CRC patients [11, 17]. The pseudogene SUCLG2P2 along with two other genes (SUCLG2 and ATIC) were introduced as potential prognostic markers for colon cancer, aiding in predicting patient outcomes and tailoring treatment strategies [24]. Another report highlighted the association of lower levels of MT1DP with inferior OS and disease‐free survival (DFS) rates in CRC patients, indicating its potential as an independent prognostic marker [27].

### 3.5 Diagnostic

Pseudogenes are gaining recognition for their diagnostic potential in CRC. Dysregulated expression patterns observed in CRC suggest their utility as biomarkers. Their distinct expression profiles in CRC tissues compared to healthy tissues highlight their appeal as diagnostic biomarkers [21, 22, 23].

The pseudogene MYLKP1, derived from MYLK which encodes myosin light chain kinase (MLCK), exhibits high expression in lung and colon cancer cell lines and tissues but is absent in normal lung or colon tissue. The promoter activity of MYLKP1 is low in normal bronchial epithelial cells but significantly elevated in lung adenocarcinoma cells. Genotyping studies conducted by Lynn et al. have identified two single nucleotide polymorphisms (SNPs), rs12490683 and rs12497343, associated with an increased risk of colon cancer in African Americans compared to controls. These SNPs are found to further enhance the promoter activity of MYLKP1 compared to the wild type, indicating that MYLKP1 functions as a colon cancer‐promoting pseudogene, with its genetic variants exacerbating colon cancer risk specifically in African American populations [23]. The gene POU5F1, responsible for encoding a transcription factor, produces two different versions called transcripts 1 and 2, alongside six pseudogenes. Transcript 1 is crucial in controlling cellular pluripotency and self‐renewal. Among the pseudogenes, POU5F1P1 located on chromosome 8q24 is particularly notable. It shares a high similarity (95%) with isoform 1 of POU5F1 and is positioned near a genetic variant (rs6983267) strongly linked to increased prostate and colon cancer risk. Additionally, POU5F1P1 is situated within amplified regions found in various human cancers. Prior research using expressed sequence tags indicates that POU5F1P1 is indeed expressed. Panagopoulos and colleagues demonstrated that the putative POU5F1P1 protein localizes in the nucleus, functions as a transcriptional activator, and regulates gene expression similarly to isoform 1 of POU5F1. However, POU5F1P1 exhibits weaker activation potential compared to isoform 1, possibly due to amino acid differences [33]. Zhong et al. identified specific long non‐coding RNAs (lncRNAs) and pseudogenes unique to CRC. They constructed a network of ceRNAs consisting of 10 lncRNAs, 5 pseudogenes, 122 mRNAs, and 39 miRNAs. Within this network, they found that H19, a lncRNA, increases the expression of cancer‐related mRNAs by competitively binding to various miRNAs, thereby activating the PI3K–Akt signaling pathway and promoting CRC progression. Cox regression and correlation analyses revealed associations between H19 and other ceRNAs in the network with poor prognosis and clinical parameters such as tumor grade and metastasis. Knockdown experiments targeting H19 resulted in reduced levels of oncogenic proteins MET, ZEB1, and COL1A1 in vitro. Additionally, other ceRNAs within the network, including the pseudogene RPLP0P2, were identified as potential contributors to cancer progression by modulating mRNA expression levels through miRNA binding. This ceRNA network sheds light on the roles of transcriptomes, particularly non‐coding RNAs, in CRC pathogenesis, offering insights for early diagnosis and therapeutic strategies [21]. NMRAL2P emerges as a newly recognized functional pseudogene for NMRAL1, influencing NQO1 regulation. Sulforaphane (SFN), a dietary component, activates the Nrf2 signaling pathway to bolster antioxidant activities and carcinogen detoxification, elevating genes crucial for these processes. Transcriptomic analysis in SFN‐exposed colon cancer cells revealed the non‐coding RNA Loc344887, regulated by Nrf2 and aiding NQO1 activity, identified as NMRAL2P. This pseudogene shares 62% homology with NMRAL1 and is situated at 3q27.2. According to data retrieved from TCGA, NMRAL2P exhibits notably lower expression in human CRCs than in normal tissues, suggesting its potential as an emergent tumor suppressor biomarker [16]. Additionally, a significant correlation between elevated plasma levels of PCNAP1 and diminished survival in patients was noted. Further comparative analyses involving healthy controls, gastric cancer (GC), and CRC groups revealed significantly higher PCNAP1 levels in hepatocellular carcinoma patient plasma. These findings suggest the potential utility of PCNAP1 as a novel diagnostic tool for HCC, gastric cancer, and CRC [19].

Pseudogenes have shown promising results as diagnostic markers in CRC, offering a new frontier in early detection and disease monitoring. By understanding their unique expression patterns in CRC, researchers can develop diagnostic tests that identify the presence of cancer and allow for precise staging.

### 3.6 Therapeutic

Three key pseudogenes that offer novel insights into CRC biology, are represented as promising targets for future therapeutic strategies. CTNNAP1, found downregulated in 70% of CRC tumors, correlates with advanced TNM staging and acts as a ceRNA, modulating CTNNA1 expression through miRNA‐141 decoy activity. This mechanism influences CRC cell growth and tumorigenesis, presenting a target for therapeutic intervention [5]. NMRAL2P, another pseudogene identified in SFN‐exposed colon cancer cells, is regulated by the Nrf2 pathway and enhances antioxidant activity and carcinogen detoxification. Its lower expression in human CRCs, compared to normal tissue, suggests its role as a tumor suppressor [16]. Lastly, DUXAP8, upregulated in CRC, promotes cell growth and migration, contributing to the EMT process. It functions as a ceRNA, sequestering miR‐577 and regulating RAB14 expression, an oncogene associated with CRC metastasis. Silencing DUXAP8 triggers apoptosis and reduces invasive capabilities, reinforcing its role in CRC development and indicating its therapeutic potential [11, 17]. Future treatment could potentially use and target these pseudogenes to develop novel therapies for patients with CRC.

## 4 Discussion

Pseudogenes, once thought to be non‐functional remnants of the genome, are now recognized as important contributors to cancer development. In colorectal cancer, they are emerging as key regulators of tumor growth and promising targets for precision medicine [4, 11, 34]. This systematic review outlined the revolutionary role of pseudogenes in CRC. Pseudogenes significantly influence colorectal cancer by regulating tumor growth, invasion, and angiogenesis through ceRNA activity, DNA methylation, and chromatin remodeling. Their dual role as tumor promoters or suppressors highlights their complexity. They show promise as biomarkers and drug targets, but further clinical validation is needed.

SUMO1P3 promotes colon cancer by enhancing proliferation, modulating the cell cycle, activating oncogenic pathways, and driving angiogenesis, while its reduction suppresses tumor growth and metastasis [28]. SUMO1P3 is highly expressed in breast cancer tissues and linked to tumor progression and poor survival. Its knockdown reduces cancer cell proliferation, migration, and invasion. SUMO1P3 acts by binding and suppressing the tumor suppressor miR‐320a, promoting tumor growth. These results highlight SUMO1P3 as a potential diagnostic and therapeutic target in breast cancer [35].

The ceRNA network involving RPLP0P2 has been shown to regulate mRNA expression by interacting with 15 specific miRNAs, highlighting the crucial role of non‐coding RNAs in colorectal cancer progression, diagnosis, and treatment [21]. RPLP0P2 is upregulated in CRC and promotes tumor progression by sponging miR‐129‐5p. Downregulating RPLP0P2 inhibits CRC cell viability, migration, and invasion, alters EMT markers, and suppresses tumor growth in vivo. miR‐129‐5p targets ZBTB20, and its inhibition reverses the effects of RPLP0P2 downregulation, while ZBTB20 overexpression counteracts miR‐129‐5p's tumor suppression. This highlights the RPLP0P2/miR‐129‐5p/ZBTB20 axis as a potential therapeutic target in CRC [36].

A recent study revealed that CDCP1 enhances CRC metastasis by regulating ROCK1, a serine/threonine. CDCP1 influences ROCK1 expression, promoting changes in ROCK1 downstream targets that facilitate CRC cell migration and invasion. Notably, elevated ROCK1 mRNA levels were associated with advanced TNM stages in CRC patients [20]. TRIM40 expression is reduced in CRC tissues and linked to better prognosis. It suppresses CRC cell proliferation by binding and ubiquitinating ROCK1, leading to its degradation and destabilization of c‐Myc. Lower c‐Myc levels relieve repression of p21, increasing p21 expression and causing G0/G1 cell cycle arrest [37].

Elevated plasma PCNAP1 correlates with poor survival and is significantly higher in HCC, GC, and CRC, suggesting its potential as a diagnostic biomarker [19]. CNAP1 is upregulated in both HBV‐infectious and noninfectious HCC tissues and is associated with poor patient survival. PCNAP1 promotes HCC cell proliferation by acting as a ceRNA that sponges miR‐340‐5p, which directly inhibits ATF7 expression. The PCNAP1/miR‐340‐5p/ATF7 signaling pathway is linked to poor prognosis in HCC, highlighting its potential as a therapeutic target [38].

AOC4P is downregulated in CRC and linked to advanced stage, metastasis, and poor survival. Its overexpression inhibits proliferation, migration, invasion, and EMT in CRC cells [25]. AOC4P is overexpressed in gastric tumors and linked to poor survival and lymphovascular invasion. Knockdown of AOC4P inhibits gastric cancer cell proliferation, migration, invasion, and promotes apoptosis in vitro, while reducing tumor growth in vivo. It regulates EMT by decreasing vimentin and MMP9 and increasing E‐cadherin levels. Clinical samples confirmed the association between AOC4P and EMT markers, highlighting its role in gastric cancer progression [39].

RP9P and PLEKHA8P1 were identified as potential prognostic biomarkers for CRC [4]. RP9P was significantly overexpressed in CRC tissues and cells. Its knockdown reduced CRC cell viability and suppressed tumorigenesis in a xenograft model. Mechanistically, RP9P functioned as a ceRNA by sponging miR‐133a‐3p, thereby preventing miR‐133a‐3p from targeting FOXQ1. This regulation led to increased FOXQ1 expression, which RP9P positively controlled. Furthermore, miR‐133a‐3p downregulation counteracted the tumor‐suppressive effects of RP9P knockdown, highlighting the RP9P/miR‐133a‐3p/FOXQ1 axis as a critical pathway in CRC progression [40]. Bioinformatics analysis of the TCGA LIHC dataset identified prognostic pseudogenes, including PLEKHA8P1. Experimental validation showed that PLEKHA8P1 and its parental gene PLEKHA8, a known oncogene, promote tumor progression in colorectal and liver cancers. Their dysregulated expression also contributes to 5‐FU chemoresistance in HCC [41].

DUXAP10 knockdown inhibits CRC cell proliferation, induces apoptosis, and increases G0/G1 arrest, suppressing tumor growth in vivo. Mechanistically, it interacts with LSD to downregulate tumor suppressors p21 and PTEN, promoting CRC progression and highlighting its potential as a therapeutic target [5]. DUXAP10 is the only pseudogene significantly overexpressed across all four GEO datasets and is frequently upregulated in multiple cancers, including liver hepatocellular carcinoma, bladder cancer, and esophageal cancer. High DUXAP10 expression correlates with poor prognosis in GC patients. Knockdown of DUXAP10 inhibits GC cell proliferation, migration, and invasion. Mechanistically, DUXAP10 interacts with PRC2 and LSD1 to transcriptionally repress LATS1 expression while binding to HuR to stabilize β‐catenin mRNA, increasing its protein levels post‐transcriptionally [42].

Elevated DUXAP8 expression has been identified as an indicator of unfavorable prognosis in colon cancer. DUXAP8 is significantly upregulated in CRC tissues compared to normal samples. Its overexpression promotes CRC cell proliferation, invasion, and EMT, while its knockdown induces apoptosis and reduces metastatic potential. Silencing DUXAP8 increases E‐cadherin and decreases vimentin, snail, and N‐cadherin expression, indicating EMT suppression. Mechanistically, DUXAP8 interacts with EZH2 to repress E‐cadherin expression via H3K27me3 modification [11, 17]. DUXAP8 was found to be significantly overexpressed in ovarian cancer (OCa) tissues and cell lines, and its high expression correlated with poor patient prognosis. Mechanistically, DUXAP8 was shown to bind to and negatively regulate microRNA‐29a‐3p, which was identified as a critical downstream target mediating DUXAP8's effects on OCa cell proliferation and migration [43].

It was demonstrated that lower MT1DP levels are associated with poorer OS and DFS in CRC, suggesting its role as an independent prognostic marker [27]. MT1DP, a lncRNA, enhances erastin‐induced ferroptosis in non‐small cell lung cancer (NSCLC) by downregulating NRF2 through stabilizing miR‐365a‐3p, leading to increased reactive oxygen species (ROS), malondialdehyde (MDA), ferrous iron, and decreased glutathione (GSH). Folate‐modified liposomes co‐delivering erastin and MT1DP (E/M@FA‐LPs) improve drug delivery and boost ferroptosis. In vivo, these liposomes effectively inhibit tumor growth, highlighting the MT1DP/miR‐365a‐3p/NRF2 pathway as a promising NSCLC therapeutic target [44].

NMRAL2P, regulated by the Nrf2 pathway, enhances antioxidant activity and carcinogen detoxification. Its reduced expression in CRC suggests a tumor‐suppressive role [16]. The lncRNA NMRAL2P is linked to oxidative stress regulation in head and neck tumors. In vitro studies revealed its oncogenic role, as it enhances lactic acid and superoxide dismutase levels while decreasing ROS and malondialdehyde production. NMRAL2P facilitates GPX2 transcription by interacting with the transcription factor Nrf2 and stabilizes ENO1, a key glycolytic enzyme, by preventing its degradation through direct binding [45].

The largest isoform of MLL3 is transcribed from a CpG island–linked promoter homologous to the pseudogene psiTPTE22, whose promoter is hypermethylated in CRCs and associated with aging in normal epithelium [22]. Liang et al. identified psiTPTE22‐HERV, a human‐specific gene derived from the 5′ region of the TPTE pseudogene psiTPTE22, containing a 3.8‐kb human endogenous retrovirus (HERV) element. This element enables independent expression, producing three alternatively spliced transcripts, including a 402‐nt ORF encoding a 15‐kDa protein. The ORF is upregulated in normal tissues such as kidney, liver, stomach, and lung, but downregulated in corresponding tumors. Located near the centromere of chromosome 22 with a GC‐rich promoter, psiTPTE22‐HERV is epigenetically silenced in cancers through DNA methylation, and its expression can be reactivated using DNA methylation and histone deacetylase inhibitors [46].

MYLKP1, a partial duplicate of the MYLK gene encoding smMLCK, shares ~89.9% promoter homology but shows low activity in normal cells and high activity in lung adenocarcinoma cells. It is strongly expressed in cancer, while smMLCK is downregulated, with a 19.5‐fold decrease in colon carcinoma. Mechanistically, MYLKP1 suppresses smMLCK expression by reducing RNA stability, promoting cancer cell proliferation, highlighting its role in tumorigenesis. Moreover, Genotyping by Lynn et al. identified two SNPs (rs12490683 and rs12497343) that enhance MYLKP1 promoter activity and are linked to increased colon cancer risk in African Americans, suggesting MYLKP1 as a cancer‐promoting pseudogene with population‐specific genetic risk factors [23, 47].

RAB9BP1, ANKRD20A9P, CXADRP2, LOC407835, UQCRBP1, and HMGB3P1 are prognostic pseudogenes in CRC [29]. However, the mechanisms by which these pseudogenes contribute to the development of CRC or other cancers are not well‐studied. Nonetheless, several articles were identified that analyze the expression status of these genes in CRC or other types of cancer. It was found that deletions in the RAB9BP1, LOC101928523, and MALRD1 genes were associated with non‐cancer patients who had a family history of cancer (FHC). The results suggest that individuals with a FHC are at a higher risk of developing cancer, potentially due to inheriting these three deleted genes [48]. A group of lncRNAs found to be upregulated in prostate cancer patients through RNA‐Seq analysis were subsequently examined for their interactions with several proteins targeted by the androgen receptor. Among these, three lncRNAs—SCARNA10, NPBWR1, and ANKRD20A9P—were identified as commonly interacting with the targeted proteins [49]. Druliner et al. demonstrated that CXADRP2 was among the top 10 genes with genetic aberrations in blood samples from normal colon tissue of proficient mismatch repair (pMMR) CRC patients [50]. It was also revealed that LOC407835 is upregulated in breast cancer samples [51]. SFXN4 is upregulated in HCC and linked to poor prognosis, tumor progression, and immune infiltration. Its knockdown reduces tumor growth, indicating therapeutic potential. UQCRBP1, positively co‐expressed with SFXN4, is also likely upregulated in HCC, suggesting a shared role in cancer progression [52]. Moreover, Xue and colleagues reported that HMGB3P1 is upregulated in CRC tissues compared to the corresponding non‐tumor tissues [53].

## 5 Limitations

Despite this overview demonstrating the advancements made in the understanding of pseudogene roles in CRC,  various limitations should be noted. Due to the limited number of studies on pseudogenes in CRC, especially in humans, more research on human samples is needed to validate the findings for clinical usage. Second, the genetic and epigenetic diversities driving CRC heterogeneity pose a challenge in identifying universal pseudogene biomarkers. Research in the future should aim to make clear the roles of pseudogenes in a tissue‐specific and disease‐stage‐specific manner so that their potential can be realized in diagnostic and prognostic settings. The advancement of reliable technologies for pseudogene detection and modulation should be another direction for future research. While CRISPR‐Cas9 among other techniques [i.e., RNA interference (RNAi)] holds great promise for selective targeting of pseudogenes in vivo,  such application in clinical settings is still in its infancy. Combining pseudogene profiling with other omics data—like genomics, transcriptomics, and proteomics—can give a clearer picture of their roles in colorectal cancer and related pathways. This approach may help discover new targets for therapy.

## 6 Future Prospect

Research on pseudogenes in CRC highlights their involvement in both promoting and inhibiting tumorigenesis through interactions with miRNAs and signaling pathways, with in vitro studies shedding light on their biological functions. Future work is recommended to explore pseudogenes as therapeutic targets to develop innovative treatments. For example, a 2019 invention relates to analgesic treatments aimed at reducing pain through the inhibition of the fatty‐acid amide hydrolase (FAAH‐OUT) pseudogene [54]. This concept is based on a case report of a 66‐year‐old female with a history of painless injuries and rapid healing. She was found to have a genetic cause for her pain insensitivity, including a microdeletion in the FAAH‐OUT pseudogene and a functional SNP in the FAAH gene. These genetic factors resulted in reduced FAAH expression and activity, leading to elevated endocannabinoid levels in her blood, indicating enhanced endocannabinoid signaling. The study emphasizes the role of FAAH‐OUT as a novel pseudogene and suggests that targeting FAAH‐OUT could provide a new approach to pain management, potentially improving treatments for postoperative and chronic pain [55]. Additionally, their diagnostic and prognostic potential in CRC could lead to breakthroughs in creating diagnostic tools and markers, particularly valuable in stage II CRC, where the use of adjuvant chemotherapy remains debated. Deepening our understanding of pseudogenes will enhance CRC diagnostics and foster the creation of targeted therapies, ultimately improving patient outcomes.

Nowadays, AI is increasingly being used in pseudogene research. One promising approach is the application of graph‐based deep learning models, such as Pseudo2GO. This approach leverages the functional similarity between pseudogenes and their parent coding genes to predict the functions of pseudogenes. By constructing a sequence similarity graph that links pseudogenes with their corresponding coding genes, Pseudo2GO incorporates various node attributes, including gene expression profiles, microRNA interactions, protein–protein interactions (PPIs), and genetic interactions. Through the use of graph convolutional networks (GCNs), the model propagates these attributes across the graph, enabling the classification of pseudogenes based on their predicted functions. This AI‐driven method has shown remarkable success, outperforming traditional techniques and achieving state‐of‐the‐art performance, especially in the M‐AUPR metric. By employing AI, this method significantly advances the understanding of pseudogene functions, offering a promising tool for genomic research [56]. The use of AI in genomic analysis has become crucial for addressing challenges such as pseudogene‐induced variant calling errors. Processed pseudogenes, created by the reverse transcription of mRNA and subsequent integration into the genome, are problematic due to their high sequence similarity to parental genes. This similarity can cause variant calling errors, as sequences from pseudogenes may be misattributed to parent genes, complicating accurate variant identification. Hence, AI‐driven approaches were employed to analyze 30x human whole‐genome sequencing data (*n* = 13 307) using popular germline variant callers, including GATK‐HC, DRAGEN, and DeepVariant. The results revealed that pseudogenes can interfere with variant calling, leading to false positives in identifying clinically relevant variants. Among the methods tested, DeepVariant, an AI‐based variant caller, was the most effective at correcting these errors [57]. Consequently, AI plays an important role in cancer research by predicting the functions of pseudogenes and improving the accuracy of variant detection, thereby enhancing genetic analysis precision and advancing diagnostics and treatment strategies.

## 7 Conclusion

This systematic review explores the significant roles that pseudogenes play in colorectal cancer (CRC), illustrating a paradigm shift from viewing them as evolutionary relics to recognizing their active role in disease progression. Key pseudogenes like DUXAP8, SUCLG2P2, and SUMO1P3 are instrumental in CRC tumorigenesis, influencing critical cellular functions such as proliferation, migration, invasion, and angiogenesis. The study identifies various pseudogenes with diagnostic and prognostic potential, including MYLKP1 and POU5F1P1. Additionally, CDCP1, DUXAP8, SUCLG2P2, and MT1DP are recognized as prognostic indicators, offering insights into clinical outcomes and supporting tailored therapeutic approaches for CRC management. The potential of pseudogenes like CTNNAP1, NMRAL2P, and DUXAP8 as therapeutic targets underscores their significance in personalized medicine, guiding patient‐specific treatments. The correlation between pseudogene expression and clinicopathological parameters strengthens the case for further research into the regulatory mechanisms of pseudogenes, which could ultimately refine diagnostic precision and improve personalized care in CRC treatment.

## Author Contributions

Conceptualization: Ali Asadzadeh and Zahra Salehi. Methodology: Zahra Salehi and Parmida Bagheri. Investigation: Parmida Bagheri, Ali Asadzadeh, Alireza Zangooie, and Zahra Salehi. Writing – original draft: Ali Asadzadeh, Alireza Zangooie, and Parmida Bagheri. Writing – review and editing: Zahra Salehi, Alireza Zangooie, and Parmida Bagheri. Illustration: Zahra Salehi, Alireza Zangooie, Ali Asadzadeh and Helia Zangooie. Project administration: Zahra Salehi. Supervision: Zahra Salehi. All authors read and approved the final version of the manuscript.

## Ethics Statement

The authors have nothing to report.

## Consent

The authors have nothing to report.

## Conflicts of Interest

The authors declare no conflicts of interest.

## Supporting information


**Table S1.** Search queries and databases.

**Table S2.** Summary of pseudogenes identified as potential diagnostic, prognostic, or therapeutic biomarkers in colorectal cancer across the included studies.

## Data Availability

This review is based on previously published studies, and all relevant data sources are cited within the manuscript. Any additional analyses or supporting information are provided in the supplementary files.
