# Supplementary material for: Pseudogenes as Potential Diagnostic, Prognostic and Therapeutic Biomarkers in Colorectal Cancer: A Systematic Review
Source: Cancer Rep (Hoboken). 2025 Jun 24;8(6):e70263. doi: 10.1002/cnr2.70263 (PMC12187983; doi:10.1002/cnr2.70263)
Supplement: Supplementary file 1 — Table S1. Search queries and databases. [file CNR2-8-e70263-s002.docx]

| **Database** | **Search Query** | **Number of extracted studies** |
| --- | --- | --- |
| PubMed | #1: "Pseudogene"[Title/Abstract] OR "Processed Gene"[Title/Abstract] OR "Processed Genes"[Title/Abstract] OR "beta tubulin pseudogenes"[Title/Abstract] OR "beta tubulin pseudogene"[Title/Abstract] OR "beta tubulin pseudogene"[Title/Abstract] OR "beta tubulin pseudogenes"[Title/Abstract] OR "Pseudogene"[Title/Abstract] OR "Pseudogenes"[Title/Abstract] OR "Pseudogenes"[MeSH Terms]  #2: "Colorectal Neoplasm"[Title/Abstract] OR "Colorectal Neoplasms"[Title/Abstract] OR "Colorectal Tumor"[Title/Abstract] OR "Colorectal Cancer"[Title/Abstract] OR "Colorectal Cancers"[Title/Abstract] OR "Colorectal Carcinoma"[Title/Abstract] OR "Colorectal Carcinomas"[Title/Abstract] OR "Colorectal Neoplasms"[MeSH Terms]  #3: #1 AND #2 | 92 |
| Web of Science | #1: (((ALL=("Colorectal Neoplasm"*)) OR ALL=("Colorectal Tumor"*)) OR ALL=("Colorectal Cancer"*)) OR ALL=("Colorectal Carcinoma"*)  #2: (((ALL=(Pseudogene)) OR ALL=("Processed Gene"*)) OR ALL=("beta-Tubulin Pseudogene"*)) OR ALL=("beta Tubulin Pseudogene"*)  #3: #1 AND #2 | 82 |
| Scopus | TITLE-ABS-KEY(pseudogene) OR TITLE-ABS-KEY("Processed Gene") OR TITLE-ABS-KEY("beta Tubulin Pseudogene"*) AND TITLE-ABS-KEY("colorectal cancer") OR TITLE-ABS-KEY("Colorectal Neoplasm"*) OR TITLE-ABS-KEY("Colorectal Tumor"*) OR TITLE-ABS-KEY("Colorectal Carcinoma"*) | 155 |

**Table S1.** Search queries and databases
